# Room temperature valley polarization via spin selective charge transfer

**DOI:** 10.1038/s41467-023-40967-7

**Published:** 2023-08-26

**Authors:** Shreetu Shrestha, Mingxing Li, Suji Park, Xiao Tong, Donald DiMarzio, Mircea Cotlet

**Affiliations:** 1 Center for Functional Nanomaterials, Brookhaven National Laboratory, Upton, NY 11973 USA; 2 Northrop Grumman Corporation, One Space Park, Redondo Beach, CA 90278 USA

**Keywords:** Two-dimensional materials, Excited states

## Abstract

The two degenerate valleys in transition metal dichalcogenides can be used to store and process information for quantum information science and technology. A major challenge is maintaining valley polarization at room temperature where phonon-induced intervalley scattering is prominent. Here we demonstrate room temperature valley polarization in heterostructures of monolayer MoS_2_ and naphthylethylammine based one-dimensional chiral lead halide perovskite. By optically exciting the heterostructures with linearly polarized light close to resonance and measuring the helicity resolved photoluminescence, we obtain a degree of polarization of up to −7% and 8% in MoS_2_/right-handed (R-(+)-) and left-handed (S-(-)-) 1-(1-naphthyl)ethylammonium lead iodide perovskite, respectively. We attribute this to spin selective charge transfer from MoS_2_ to the chiral perovskites, where the perovskites act as a spin filter due to their chiral nature. Our study provides a simple, yet robust route to obtain room temperature valley polarization, paving the way for practical valleytronics devices.

## Abstract (web summary)

Valleytronics is a portmanteau of ‘valley’ and ‘electronics’ and refers to the use of the valley degree of freedom, present in some materials, for encoding and processing information. Here, Shrestha et al demonstrate a room temperature valley polarization in heterostructures composed of molybdenum disulfide and a chiral lead halide perovskite, an important step in development of valleytronics.

## Introduction

**Fig. 1:**
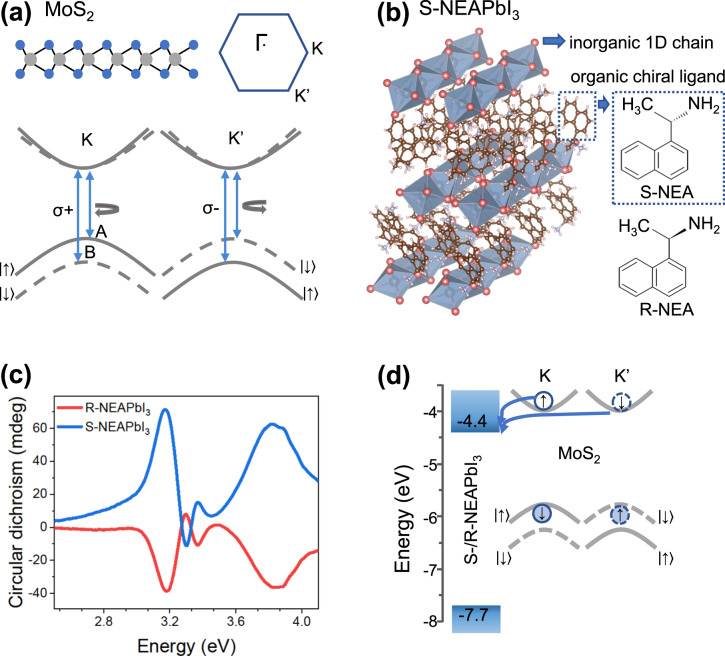
Heterostructure of monolayer MoS_2_ and 1D chiral perovskite. **a** side view of the crystal structure of a monolayer MoS_2_ showing the broken crystal inversion symmetry (Mo and S atoms shown in gray and blue, respectively). The hexagonal Brillion zone has K and K′ points at the corners. The electronic band structure at these points (bottom) has a direct bandgap with spin split valence band. A and B are exciton transitions from the conduction band to the upper and lower split valence bands, respectively. Due to optical selection rules, only K (K′) valley emits or absorbs σ+ (σ−). **b** crystal structure of chiral perovskite (S-(-)-) 1-(1-naphthyl)ethylammonium lead iodide (S-NEAPbI_3_)^24^. The inorganic 1D lead halide chain (Pb and I atoms shown in gray and red) is surrounded by chiral organic ligands. The dotted blue box shows the molecular structure of the chiral organic ligand S-1-(1-naphthyl)ethylamine (S-NEA). The molecular structure of the enantiomer R-1-(1-naphthyl)ethylamine (R-NEA) is also shown. **c** Circular dichroism spectra of thin films of R-NEAPbI_3_ (red) and S-NEAPbI_3_ (blue). **d** Energy band alignment of the heterostructure components. The electronic bands for S-NEAPbI_3_ are derived from ultraviolet photoelectron spectroscopy and UV-vis measurements. The energy is relative to the vacuum level. The bands for monolayer MoS_2_ are taken from literature^32,33^ and the valence band splitting is exaggerated for clarity. Electrons and holes are depicted as empty and filled circles, respectively.

Besides an electron’s charge and spin, the valley it occupies in momentum space can also be used to process and store information for classical or quantum computing. As the valleys are often well separated in momentum space, the valley index is robust against scattering^1^ and offers a reliable new degree of freedom. Two-dimensional (2D) materials with a honeycomb lattice such as transition metal dichalcogenides (TMDs) have two degenerate valleys, K and K′, at the corners of their hexagonal Brillion zone (Fig. 1a) and are great candidates for valleytronics. Monolayers of group VI of TMDs in 2H phase have a direct bandgap at the K point and a broken crystal inversion symmetry (D^1^_3h_) which leads to valley-dependent optical selection rules^2^. Moreover, strong spin–orbit coupling due to the heavy metal atoms splits the electronic band by hundreds of meV^3^. Since time reversal symmetry requires that the spin splitting at the two valleys is opposite, spin and valley are coupled^1^ or locked. Therefore, to change an electron’s valley, a simultaneous flip in its spin is required which makes valley index in monolayer TMDs robust.

Selectively populating a specific valley, or valley polarization which can be considered as writing or encoding data, is the first step towards valleytronics applications. The most common route used to obtain valley polarization in TMDs is via optical pumping with a circularly polarized light. Due to the valley-dependent optical selection rules, K and K′ valleys can be selectively populated with left (σ+) and right (σ−) circularly polarized light, respectively. Similarly, valley polarization can be read out by measuring the σ+ and σ− components of the emitted photoluminescence (PL)^4–8^. The valley population in TMDs can be further manipulated using strain^9^, magnetic field^10,11^, or by adding plasmonic metasurfaces^12^. Moreover, taking advantage of the layered nature of TMDs, enhanced valley polarization due to charge transfer and other proximity effects in heterostructures consisting of TMDs^13,14^, other 2D materials^15^ and magnetic materials^16^ has also been demonstrated.

Valley polarization was recently demonstrated in heterostructures combining TMDs and 2D chiral lead halide perovskites^17^. In these heterostructures, a chirality induced spin selectivity effect^18,19^ results in chiral perovskites acting as spin filters, preferentially injecting charges with one spin, depending on the handedness of the perovskite material. Since spin and valley in monolayer TMDs are coupled, the valley population is therefore controlled via selective spin injection. This elegant, yet simple way to control valley polarization does not require circularly polarized light, magnetic field, or complex device structure. However, the phenomenon was observed only at low temperature (78 K) and steadily decreased as the temperature increased, leaving no polarization at room temperature. In fact, the majority of studies mentioned here so far have been performed only at low temperatures^4–8,10,11,13,14,16^, as depolarization due to intervalley scattering becomes prominent at higher temperature^17,20^. Requirement of cryogenic temperatures makes these systems expensive, bulky, and complex. Achieving robust valley polarization in TMDs at room temperature is a challenge which needs to be addressed for practical applications of TMD-based valleytronics devices in quantum information science and technology.

Here we demonstrate room temperature valley polarization in heterostructures of monolayer MoS_2_ and one-dimensional (1D) chiral lead halide perovskite R-(+)- or S-(-)-1-(1-naphthyl)ethylammonium lead iodide (R- or S-NEAPbI_3_). We obtain a degree of polarization of up to 8% by exciting MoS_2_ with a linearly polarized laser with energy close to A-exciton resonance, at ambient conditions. We attribute the ability to observe room temperature valley polarization to a stronger chirality of the 1D perovskite system versus earlier reported 2D perovskite together with resonant excitation conditions employed here. Our results demonstrate a simple and robust method to control room temperature valley polarization in TMDs, thus paving the way for practical valleytronics devices which could change the way we store and process information using conventional charge and spin-based electronic devices.

## Results

We begin by synthesizing single crystals of R/S-NEAPbI_3_ chiral lead halide perovskites using a slow cooling method^21–23^. Powder X-ray diffraction pattern of the crystals are consistent with literature^22^ (Supplementary Fig. 1) and show the absence of impurities. Figure 1b shows the crystal structure of 1D S-NEAPbI_3_ chiral lead halide perovskite^24^. The hybrid organic-inorganic material consists of inorganic 1D chains of face-sharing lead halide octahedra surrounded by organic chiral ligands S-(-)-1-(1-naphthylammine). The organic chiral ligands form asymmetric hydrogen bonds with the inorganic framework and have been reported to transfer chirality via helical structural distortions^22^ and asymmetric electronic interactions^25^. In comparison to 2D chiral perovskites, where chiral ligands are located between 2D lead-halide sheets, the 1D chiral perovskites show a stronger chiroptical activity because the chiral ligands surrounding the lead-halide chains lead to a stronger distortion in the later systems^26,27^. The circular dichroism (CD) spectra of spin cast thin films of R- and S-NEAPbI_3_ in Fig. 1c show strong CD signals, with peaks appearing at the same energy, but with opposite signs for the two enantiomers. This confirms their preference for absorption of light with opposite helicities. The bisignate signal at about 3.2 eV corresponds to exciton absorption (Supplementary Fig. 2) and is in agreement with literature^26^.

Weak van der Waals interactions between the organic ligands allows the chiral perovskite crystals to be mechanically exfoliated into thin flakes with smooth surfaces^28–30^ (confirmation by atomic force microscopy (AFM) in Supplementary Fig. 3). To make heterostructures, MoS_2_ flakes were mechanically exfoliated on Si/SiO_2_ substrate and monolayers were identified using AFM and Raman micro-spectroscopy (Supplementary Fig. 4). Next, chiral perovskite flakes roughly 500 nm thick were stacked on top of the monolayer MoS_2_ using a dry transfer method^31^. Electronic energy bands measured using ultraviolet photoelectron spectroscopy (UPS) show that the chiral perovskites have a valence band maxima at −7.7 eV relative to the vacuum level (Supplementary Fig. 5). Considering a bandgap of 3.3 eV estimated from the UV-Vis absorption data (Supplementary Fig. 2), the conduction band minima is at about −4.4 eV relative to the vacuum level, which provides a type II (staggered) energy band alignment between the perovskite and monolayer MoS_2_ (Fig. 1d).

### Photoluminescence measurements

**Fig. 2:**
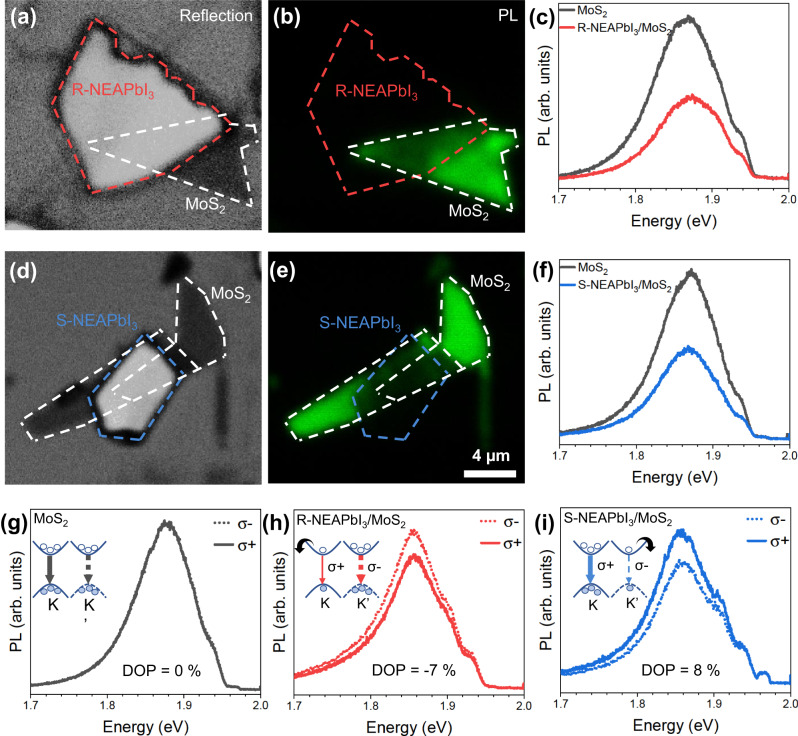
Photoluminescence of MoS_2_/1D chiral perovskite heterostructures. **a** Optical reflection image and **b** PL map of R-NEAPbI_3_/MoS_2_ heterostructure. **c** PL spectra from MoS_2_ only region (black) and overlap region (red) of R-NEAPbI_3_/MoS_2_ heterostructure. **d** Optical reflection image and **e** PL map of S-NEAPbI_3_/MoS_2_ heterostructure. **f** PL spectra from monolayer MoS_2_ only region (black) and overlap region (blue) of S-NEAPbI_3_/MoS_2_ heterostructure. The dotted white, red, and blue lines outline the monolayer MoS_2_, R-NEAPbI_3_ flake and S-NEAPbI_3_ flake, respectively. Polarization resolved PL spectra of **g** monolayer MoS_2_, **h** R-NEAPbI_3_/MoS_2_, and **i** S-NEAPbI_3_/MoS_2_. The solid lines and dotted lines show left (σ+) and right (σ−) circularly polarized components of the PL, respectively. The inset shows schematics of electronic bands at K and K′ valley with respective optical selection rules. The arrows indicate preferential electron transfer from specific valleys in MoS_2_ to R- and S-NEAPbI_3_ chiral perovskite, respectively. Electrons and holes are depicted as empty and filled circles.

We optically excited the heterostructure with a linearly polarized laser of 1.96 eV energy and measured the emitted photoluminescence (PL) in a confocal microscope (see “Methods” for details). As the chiral perovskite has a bandgap of 3.3 eV (Supplementary Fig. 2), we expect negligible absorption of the laser light by this component of the heterostructure. Figure 2a, b shows the reflection and PL maps of a R-NEAPbI_3_/MoS_2_ heterostructure, where white and red dash lines outline the monolayer MoS_2_ and the chiral perovskite flake R-NEAPbI_3_, respectively. The overlap region of the heterostructure demonstrates a quenched PL signal in comparison to the pristine monolayer MoS_2_ region. The PL spectra in Fig. 2c shows that both monolayer MoS_2_ region and the overlap region of the heterostructure have peaks at 1.86 eV, which we relate to the A-exciton optical transition from the conduction band to the upper split valence band in MoS_2_. However, the PL spectrum from the overlap region is much lower in intensity. We obtained similar results also for the S-NEAPbI_3_/MoS_2_ heterostructure which are shown in Fig. 2d–f. Although there is some variation in the intensity of PL emission from point to point on a sample, the PL from the overlap regions is quenched, on average, 50% in comparison to the pristine monolayer region. Looking at the type II energy band alignment of the heterostructure components shown in Fig. 1d, photoinduced electron transfer is expected to occur from the conduction band of the photo-excited monolayer MoS_2_ to R-NEAPbI_3_. This photoinduced charge transfer competes with radiative recombination in MoS_2_, resulting in a quenched PL of MoS_2_. Emission at low energy from interlayer excitons, which have been reported in TMD/2D perovskite heterostructures^20,34^, was absent in the spectral range probed (1.3–2 eV). Therefore, in this work we focus on only MoS_2_ emission.

The polarization resolved PL signal of monolayer MoS_2_ (Fig. 2g) shows that the left (σ+) and right (σ−) circularly polarized components of the PL spectrum are identical in peak position, shape, as well as intensity. The R-NEAPbI_3_/MoS_2_ heterostructure (Fig. 2h), however, has a higher PL emission peak intensity for σ− (dotted line), while the S-NEAPbI_3_/MoS_2_ heterostructure (Fig. 2i) has a higher PL emission peak intensity for σ+ (solid line). Because we optically pump both K and K′ valleys equally with a linearly polarized light, for monolayer MoS_2_, the PL emitted resulting from radiative recombination from the K and K′ valleys is expected to be similar, as observed here in Fig. 2g. The PL emission differences observed for the heterostructures can be explained taking into account the electronic band structure of MoS_2_ (Fig. 1a), where spin–orbit coupling splits the valence band maxima into two bands separated by about 150 meV^3^. Excitation with the 1.96 eV laser light promotes electrons only from the upper spin split valence band, associated with the A-exciton, and this populates the conduction band with spin up electrons in the K valley and spin down electrons in the K′ valley. The photo-excited electrons in the conduction band of MoS_2_ are then driven by the type II (staggered) heterostructure to the conduction band of chiral perovskite (Fig. 1d). However, here the chiral perovskite acts as a spin filter: R-NEAPbI_3_ preferentially accepts spin up electrons, while S-NEAPbI_3_ preferentially accepts spin down electrons. This creates a population imbalance in the two valleys, resulting in a higher σ− PL emission intensity in the R-NEAPbI_3_/MoS_2_ heterostructures and vice versa in the S-NEAPbI_3_/MoS_2_ heterostructures (as shown in insets in Fig. 2h-i). In the overlap region with bilayers MoS_2_ in Fig. 2e, we observe negligible difference between σ+ and σ− PL emission (Supplementary Fig. 6). This is expected since the inversion symmetry is preserved in bilayers and therefore spin and valley are not coupled^6^.

To quantify the chirality of the PL emission, we use the degree of polarization (*DOP*) defined as:

1 $${DOP}=\frac{I\left(\sigma+\right)-I\left(\sigma -\right)}{I\left(\sigma+\right)+I\left(\sigma -\right)}*100\%$$

where *I*(σ+) and *I*(σ−) are the peak intensities of left and right circular polarized components of the PL emission, respectively. The *DOP* for monolayer MoS_2_, R-NEAPbI_3_/MoS_2_, and S-NEAPbI_3_/MoS_2_ are 0%, −7%, and 8%, respectively. A value of *DOP* of 0% obtained for monolayer MoS_2_ confirms that artifacts due to difference in transmission of σ+ and σ− of optical components of the confocal setup, such as mirrors and lenses, are negligible and validates our measurements. In Supplementary Fig. 7, we show PL measured at different laser intensities for a set of R-NEAPbI_3_/MoS_2_ and S-NEAPbI_3_/MoS_2_ heterostructures which also showed *DOP* of −5 ± 1% and 6 ± 2%, respectively. Heterostructures transferred on hBN also demonstrated a *DOP* of −6%, confirming that the substrate does not affect the *DOP* (Supplementary Fig. 8). Time-dependent PL measurements (Supplementary Figs. 9, 10) show that the observed phenomenon is also stable over time.

**Fig. 3:**
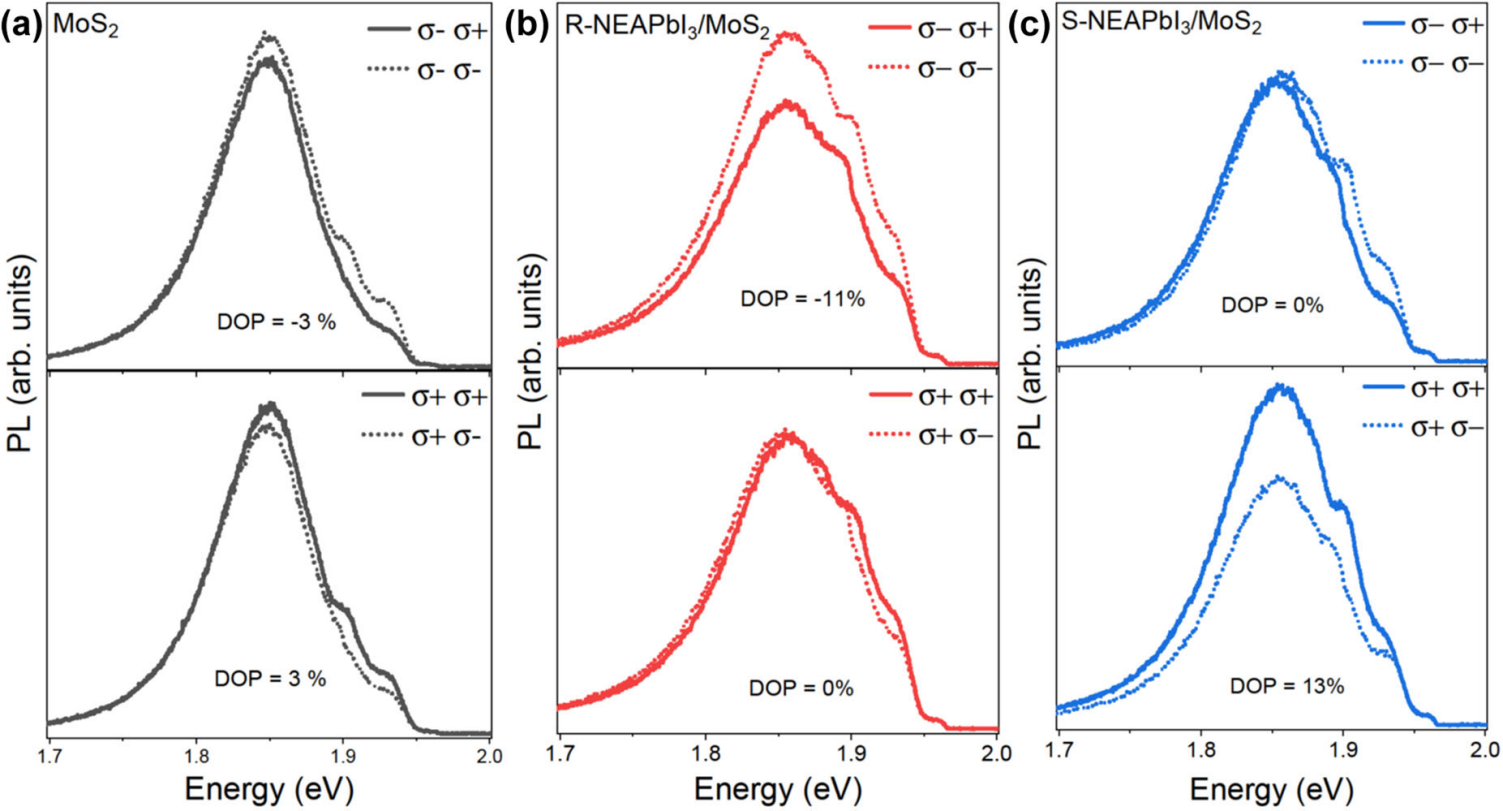
Photoluminescence with circularly polarized excitation. Polarization resolved PL spectra with circularly polarized excitation of **a** monolayer MoS_2_, **b** R-NEAPbI_3_/MoS_2_, and **c** S-NEAPbI_3_/MoS_2_. The legend indicates the polarization of excitation followed by polarization of the emission. The top and bottom plots show measurements performed with right (σ−) and left (σ+) circularly polarized excitation, respectively, at 1.94 eV. The solid lines and dotted lines show σ+ and σ− circularly polarized components of the emitted PL, respectively.

By using circularly polarized excitation we could further enhance the *DOP*. Figure 3 shows PL measurements performed with circularly polarized 1.96 eV laser. For MoS_2_ monolayer, we obtained *DOP* of −3% and 3% for excitation with σ− and σ+, respectively. The *DOP* of R-NEAPbI_3_/MoS_2_ heterostructure was −11% for σ− excitation and 0% for σ+ excitation. Similarly, the *DOP* of S-NEAPbI_3_/MoS_2_ heterostructure was 0% and 13% for σ− and σ+ excitation, respectively. These results are also consistent with spin selective electron transfer from MoS_2_ to chiral perovskite. In bare monolayer MoS_2_, the emission is higher when the polarization of excitation and detection are the same (σ+σ+ or σ−σ−) in comparison to the case when they are opposite (σ−σ+ or σ+σ−), as we pump and probe the same valley in the former case^4–8^. The *DOP* for σ+ and σ− excitation also have the same magnitude but opposite sign since the two valleys are symmetric. In R-NEAPbI_3_/MoS_2_ heterostructure, however, when K′ valley is selectively excited by σ−, σ+ emission from K valley is further reduced due to preferential transfer of spin up electrons from K valley to R-NEAPbI_3_. Therefore, the *DOP* for σ−(σ+) excitation is further enhanced while that for σ+(σ−) excitation is diminished in R-NEAPbI_3_(S-NEAPbI_3_)/MoS_2_ heterostructure in comparison to bare MoS_2_ monolayer. We note that besides the peak at 1.86 eV, the emission appears to have an additional peak at 1.89 eV. As we observed this signal also from SiO_2_ substrate measured under the same conditions, we attribute the peak to scattered laser leaking in through the filters (Supplementary Figs. 11, 12).

**Fig. 4:**
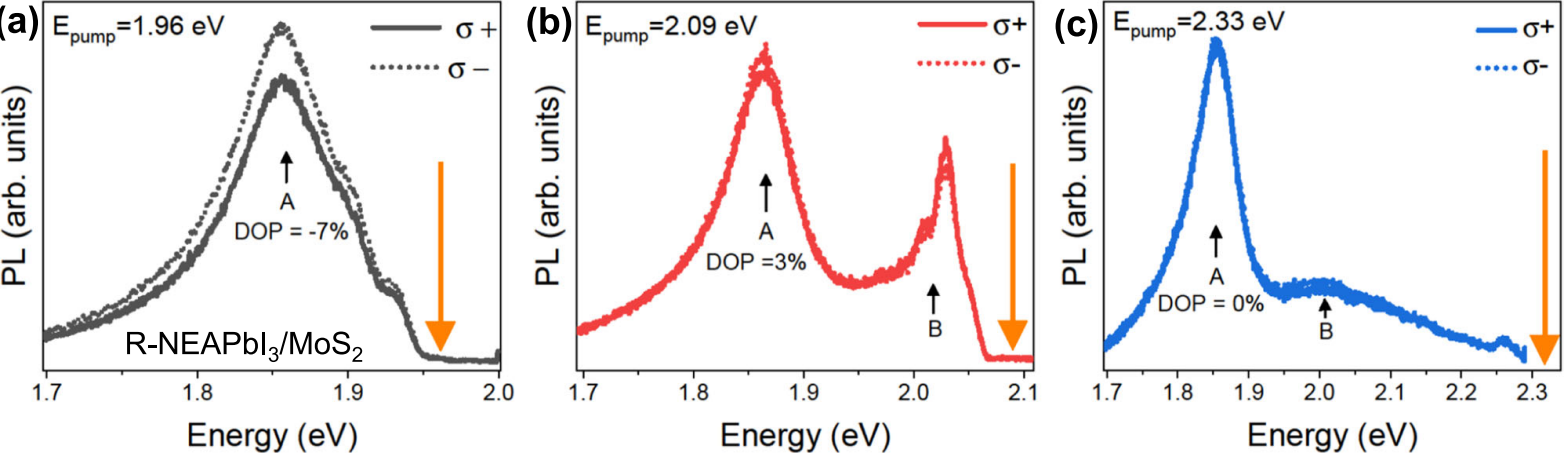
Laser excitation energy dependency of valley properties. Helicity resolved PL spectra of R-NEAPbI_3_/MoS_2_ with **a** 1.96 eV (or 633 nm), **b** 2.09 eV (or 594 nm), and **c** 2.33 eV (or 532 nm) laser excitation. The solid and dotted lines show left (σ+) and right (σ−) circularly polarized components of the PL, respectively. The orange arrows indicate the energy of the laser used for optical excitation.

The energy of optical excitation is another parameter that can be used to manipulate valley polarization in these heterostructures. Figure 4 shows polarization resolved PL spectra of the R-NEAPbI_3_/MoS_2_ heterostructure obtained with linear polarized laser excitation of 1.96 eV, 2.09 eV, and 2.33 eV in Fig. 4a–c, respectively. For the A-exciton transition, we observed a *DOP* of −7% for the 1.96 eV laser excitation which decreased to −3% for the 2.09 eV optical excitation and for the same heterostructure sample. Similarly, no polarization, i.e., a *DOP* = 0%, was observed for the 2.33 eV optical excitation. These are observations consistent with reports pointing to a lower valley polarization under non-resonant excitation conditions due to higher intervalley scattering^5,35^. We note that for 2.09 eV optical excitation, the noise from the scattered laser made it difficult to access the polarization of the B-exciton emission located at about 2.02 eV. However, for R-NEAPbI_3_/MoS_2_, we expect to observe an opposite polarization behavior for the B-exciton with 2.09 eV excitation, i.e., *DOP* values negative and positive for A- and B-exciton transitions, respectively. This is because a linearly polarized laser with an energy of 2.09 eV will excite electrons from both the upper and lower spin split valence bands, while only spin up electrons are preferentially transferred from the conduction band of monolayer MoS_2_ to R-NEAPbI_3_ in the R-NEAPbI_3_/MoS_2_ heterostructure. Due to valley contrasting spin splitting of valence bands, the resulting population imbalance in the K and K′ valleys should be opposite for A and B excitons (schematic in Supplementary Fig. 13). Therefore, by using chiral perovskites as spin filters, it is possible to simultaneously populate both the K and K′ valleys with either spin up or spin down electrons. With a circularly polarized excitation, one can manipulate only a specific valley: irrespective of the energy of the optical excitation, σ+(σ−) will populate only the K (K′) valley. However, the spin filtering strategy offers the possibility of using excitation energy as an additional parameter to manipulate valley and spin polarization.

### Transient absorption spectroscopy measurements

**Fig. 5:**
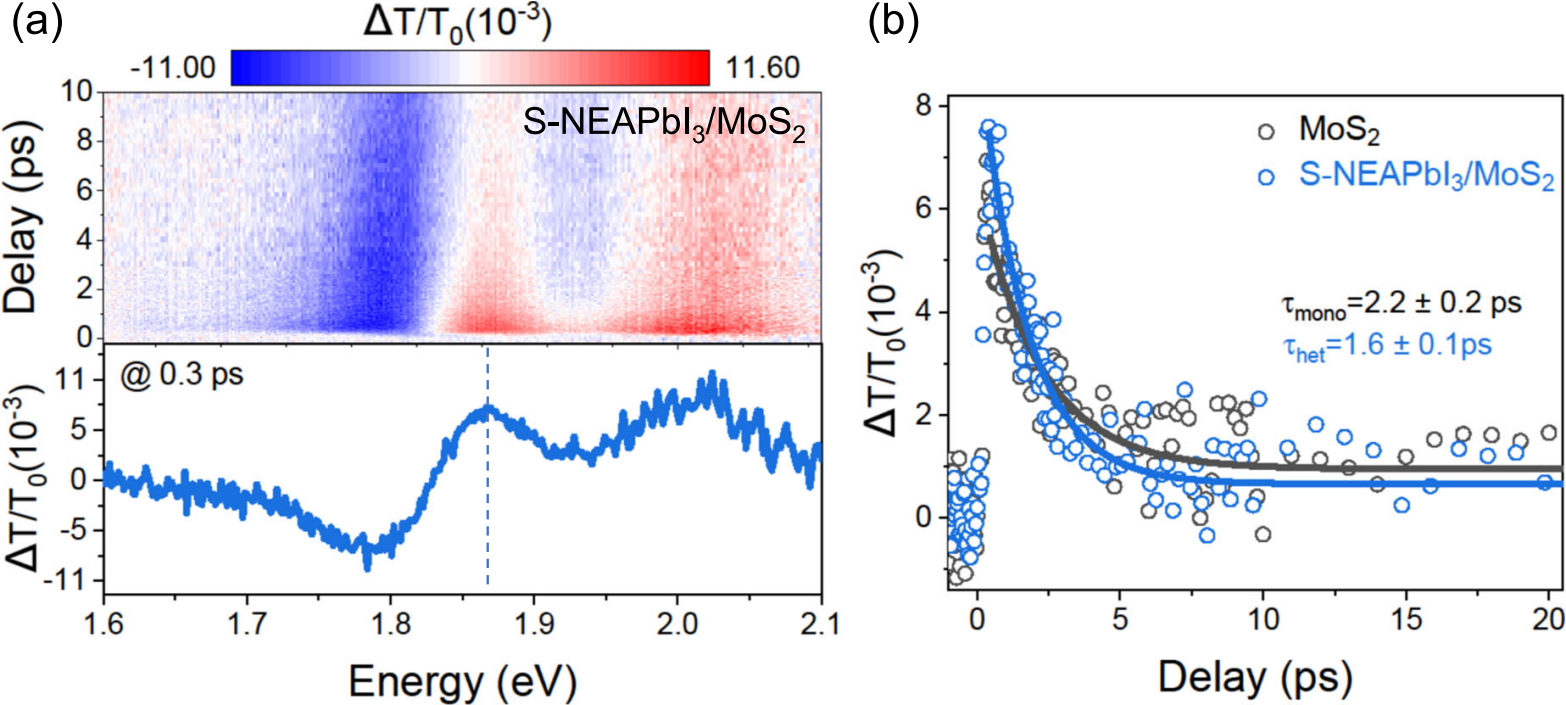
Broadband transient absorption spectra of S-NEAPbI_3_/MoS_2_ heterostructure. **a** 2D image of a differential transmission spectra (Δ*T*/*T*_0_) as a function of probe delay. The color bar shows the intensity of the Δ*T*/*T*_0_ with red and blue indicating positive and negative values, respectively. The plot at the bottom shows Δ*T*/*T*_0_ spectra at a delay of 0.3 ps. **b** Decay kinetics of monolayer MoS_2_ (black circles) and S-NEAPbI_3_/MoS_2_ heterostructure (blue circles) at 1.87 eV (shown by a dotted line in (**a**)). The solid lines are fit to mono-exponential decay function.

We obtained direct evidence for a photoinduced charge transfer in the presented heterostructures by pump-probe transient absorption spectroscopy. We optically pumped the samples at 2.48 eV (40 µJ cm^−2^ fluence, 200 fs pulse width) and measured the photo-induced changes in absorption of a white light continuum probe after a controlled time delay. Figure 5a shows the differential transmission 2D plot (top) and transient spectra at 0.3 ps delay (bottom) of the MoS_2_/S-NEAPbI_3_ heterostructure, with features at 1.8 and 2.0 eV corresponding to the A- and B-exciton transitions of MoS_2_, respectively^36–38^ (Supplementary Fig. 14). The decay of the A-exciton at 1.87 eV for the heterostructure versus the monolayer MoS_2_ is shown in Fig. 5b. Using a single exponential decay model (solid lines), we obtained carrier lifetimes of 2.2 and 1.6 ps for the monolayer MoS_2_ (*τ*_mono_) and for the heterostructure MoS_2_/S-NEAPbI_3_ (*τ*_Het_), respectively. We attribute the shorter carrier lifetime observed in the heterostructure to electron transfer from monolayer MoS_2_ to 1D chiral perovskite. Faster decays due to ultrafast charge transfer between TMDs and perovskites (as fast as 45 fs) have been previously reported^39–43^ from transient absorption and time-resolved PL measurements. We estimated a charge transfer rate, $1/{\tau }_{{{{{{{\rm{CT}}}}}}}}=1/{\tau }_{{{{{{{\rm{Het}}}}}}}}-1/{\tau }_{{{{{{{\rm{mono}}}}}}}}$, and a charge transfer efficiency, ${\Phi }_{{{{{{{\rm{CT}}}}}}}}=1-{\tau }_{{{{{{{\rm{Het}}}}}}}}/{\tau }_{{{{{{{\rm{mono}}}}}}}}$, of 1/5.9 ps^−1^ and 27%^44,45^.

## Discussion

In an ideal case, where there is good contact between the monolayer TMD/1D perovskite layers of the heterostructure and a 100% spin selectivity of the chiral perovskites, for a S-NEAPbI_3_/MoS_2_ heterostructure one expects that all the electrons from the conduction band in the K′ valley transfer to S-NEAPbI_3_, while no electrons from the K valley transfer to S-NEAPbI_3_. This ideal case would result in MoS_2_ PL quenching of 50% and a *DOP* of 100%. Although we observed roughly 50% quenching, the *DOP* value of 8% is much lower. Possible reasons can be: (1) electrons from K′ valley can recombine with holes radiatively, before being transferred to S-NEAPbI_3_; (2) electrons from K valley can be transferred to S-NEAPbI_3_ since the spin selectivity of S-NEAPbI_3_ is less than 100%; or (3) electrons from both K and K′ valleys can scatter to the opposite valley and depolarize. While depolarization due to intervalley scattering can be suppressed by lowering the temperature, requirements for cryogenic temperature would increase complexity and cost of the experiment. Alternative strategies can be adopted in the future to enable faster charge transfer and higher spin selectivity in such heterostructures. As hybrid lead halide perovskites offer large flexibility in terms of composition engineering, the energy band alignment in the heterostructure can be tuned to facilitate faster charge transfer. Similarly, more suitable chiral ligands could be designed to improve spin selectivity.

In summary, we demonstrate robust valley polarization in monolayer MoS_2_/1D chiral perovskite heterostructures at room temperature via spin selective charge transfer. By pumping with a linearly polarized laser light close to A exciton resonance and measuring helicity resolved PL, we obtain a *DOP* of 0%, −7%, and 8% for monolayer MoS_2_, MoS_2_/R-, and S-NEAPbI_3_ heterostructures, respectively. We attribute this to the chiral perovskites which act as a spin filter and control the valley population in monolayer MoS_2_ via spin selective charge transfer. We show that the polarization decreases under non-resonant excitation energy and predict that by selecting appropriate excitation energy, either spin up or spin down carriers could be selectively populated in both the valleys. Moreover, using ultrafast transient absorption spectroscopy, we present direct evidence for charge transfer from MoS_2_ to chiral perovskite. Our results provide a simple and robust route to achieve valley polarization in monolayer TMDs without the need of low temperature, magnetic field, or complex device structure, thus paving the way for practical valleytronics devices.

## Methods

### Sample preparation

Chiral lead halide perovskite single crystal was synthesized following protocols reported in literature^21–23^. In brief, equimolar amounts of R- and S-1-(1-naphthyl)ethylamine (R- and S-NEA, from Tokyo Chemical Industries) and lead iodide (PbI from Sigma Aldrich) were dissolved in HI (47% in H_2_O from Sigma Aldrich) and H_3_PO_2_ (50% in H_2_O from Sigma Aldrich). The ratio of HI:H_3_PO_2_ was 5:1. The solution was heated to 100 °C under ambient conditions and continuous stirring. Once a clear solution was obtained, the solution was slowly cooled to room temperature resulting in single crystal precipitation. The single crystals were rinsed in chloroform and dried in vacuum at 60 °C for 3 h.

Perovskite thin films used for CD spectroscopy were made by spin coating inside an Argon filled glovebox. 10 wt% perovskite solution in Dimethylformamide (DMF) was prepared. 100 µl of the precursor solution was deposited on quartz substrates cleaned in acetone and isopropanol in an ultrasonic bath for 10 min each. The substrates were spun at 4000 rpm for 30 s. Finally, the substrates were annealed at 100 °C for 30 min.

To produce monolayer MoS_2_, bulk MoS_2_ crystals (from HQ Graphene) were mechanically exfoliated using the standard “scotch tape” method. In brief, a small piece of bulk MoS_2_ was placed on a sticky tape and then the tape was folded a peeled several times to thin down the flake. Finally, the tape was pressed onto a clean Si substrate with 290 nm thick SiO_2_ and slowly peeled off. Monolayers transferred on the Si substrate were identified with an optical microscope and confirmed with Raman micro-spectroscopy and AFM.

For transmission measurements, a CVD grown monolayer of MoS_2_ on sapphire substrate (from 2D Semiconductors) was used.

Heterostructures were made with dry transfer technique^31^ using a commercial transfer stage from HQ graphene under ambient conditions. Perovskite flakes were directly exfoliated on a Polydimethylsiloxane (PDMS) stamp (from Gelpak). The PDMS stamp with perovskite was turned upside down and aligned on top of a monolayer MoS_2_. The PDMS stamp was slowly lowered and pressed on top of the target monolayer MoS_2_ flake. After the entire perovskite flake was in contact with MoS_2_, the PDMS stamp was slowly retrieved. This resulted in the perovskite flake being transferred from the PDMS stamp to the monolayer MoS_2_ flake on Si/SiO_2_ substrate. Perovskite flakes were transferred on CVD grown monolayer MoS_2_ on sapphire substrate (2D Semiconductors) for transient absorption measurements. For all other measurements mechanically exfoliated monolayer MoS_2_ on Si/SiO_2_ substrate were used.

### Characterization

Photoluminescence (PL) was measured with a home-built confocal microscope consisting of an Olympus IX81 inverted microscope equipped with a ×60 dry objective lens, NA 0.7 (Olympus America) and delivering a spatial resolution of ~0.59 µm. Samples were excited with a continuous wave monochromatic laser light of 633 nm (He Ne Melles Griot), 594 nm (He Ne Melles Griot), or 532 nm (Diode laser Sapphire, Coherent). A polarizing beam-splitter cube was used to linearly polarize the excitation beam. Appropriate laser-line optical filters were used to clean the excitation laser. The PL emitted from the sample was collected by the same objective lens. A dichroic beam-splitter mirror and a long-pass filter were used to filter out the excitation laser from the emission. For PL spectra, the emission was directed to a monochromator (Acton SpectraPro 2750) via a multimode optical fiber. A quarter waveplate and a linear polarizer were added in front of the optical fiber to separately detect the left and the right circularly polarized PL emission for helicity resolved PL measurements. Similarly, another quarter waveplate was added after the linear polarizer to create circularly polarized laser excitation. For PL mapping, the emission was spatially filtered with a 100 µm pinhole. To simultaneously measure PL and reflection images, the emission was split by a 50:50 cube and imaged onto two single-photon-counting avalanche photodiodes (MPD, Picoquant). For the PL map, an additional long-pass filter was used to filter out the laser. The photodiodes were coupled to a time analyzer (PicoHarp 300, PicoQuant) and the SymPhoTime 64 software (Picoquant) was used for data acquisition and analysis.

Transient Absorption spectroscopy (TAS) was performed with a commercial broadband transient absorption microspectrometer (Helios Fire, Ultrafast Systems). The fundamental optical output from an Ytterbium-doped Potassium-Gadolinium Tungstate (Yb:KGW) laser (PHAROS, Light conversion, 1030 nm, 195 fs, 1 kHz) was split into two parts. One part was directed to an optical parametric amplifier (Orpheus/Lyra, Light conversion) to generate a pump beam (500 nm). The other part was focused on a sapphire crystal to generate a super-continuum white-light probe beam. The time delay between the pump and probe beams was controlled using a computer-driven optical delay stage (SmartDelay Line, Ultrafast Systems). The beams were combined and focused on the sample with a ×20 objective lens (NA 0.6, Nikon). The laser spot of the pump and the probe were 10 µm and 7 µm in diameter, respectively. The transmitted probe beam was collected by another ×20 objective lens and directed to the Helios spectrometer, with experiments performed at a fluence of 40 µJcm^−2^. All measurements were performed under ambient conditions.

Power X-ray diffraction (XRD) was performed with Rigaku SmartLab Universal Diffractometer. R-SNEAPbI_3_ single crystals were crushed into fine powder using mortar and pestle. X-ray tube was operated at 40 kV and 45 mA.

Raman micro-spectroscopy was performed with a WiTec Alpha 300/Apron microscope (a part of the Quantum Materials Press facility at the Center for Functional Nanomaterials). A 532 nm laser was used for excitation in combination with a 100×objective lens.

Atomic force microscopy (AFM) was performed with an Asylum Cypher AFM ES VRS in AC tapping mode.

Circular dichroism (CD) spectroscopy of perovskite thin films on quartz substrates was measured with a Jasco J-815 CD Spectropolarimeter. Each sample was flipped and measured from both directions with the beam first hitting the (1) perovskite side (front), and (2) the quartz side (back). The average of the front and the back measurements were computed to obtain CD spectra.

Ultraviolet photoelectron spectroscopy (UPS) was carried out in an ultrahigh vacuum system with base pressure of 2 × 10^–10^ Torr equipped with a SPECS Phoibos 100, MCD-5 hemispherical energy analyzer, and an ultraviolet source UVS 10/35. He(I) at 21.2 eV radiation was used. The angle between the analyzer and UV- source was 45° and photoelectrons were collected along the sample surface normal. R-SNEAPbI_3_ single crystals were crushed into fine powder using mortar and pestle and placed on a Flag style sample plate. The spectra were calibrated vs the Fermi edge of Au (111) crystal. The work-function of the material was determined by subtracting the ultraviolet radiation energy (21.2 eV) from the secondary electron cut-off energy of UPS. The energy gap between valence band maxima to Fermi level was determined by UPS low binding energy edge to Fermi level. Bandgap between conduction and valence bands was obtained by UV-vis. Thus, the conduction band (CB) edge, valence band (VB) edge and Fermi level relative to the vacuum level was estimated as shown in Fig. 1d. Full data in SI Fig. 4.

UV-vis transmission of monolayer MoS_2_ was measured in an inverted microscope (Nikon Eclipse Ti2, New Technology & Consulting, Germany). The samples were illuminated with a broadband light source (Energetiq LDLS EQ-99) and the transmitted light was directed to a monochromator (Acton SpectraPro HRS-500) and a PIXIS Roper camera.

### Supplementary information

Supplementary Information

Peer Review File

## Data Availability

All data generated and analyzed during this study are included in this article and its supplementary information files and are available on reasonable request.
