# Supplementary material for: Room temperature valley polarization via spin selective charge transfer
Source: Nat Commun. 2023 Aug 26;14:5234. doi: 10.1038/s41467-023-40967-7 (PMC10460417; doi:10.1038/s41467-023-40967-7)
Supplement: Supplementary file 1 — Supplementary Information [file 41467_2023_40967_MOESM1_ESM.pdf]

# Supplementary Information:

# Room Temperature Valley Polarization via Spin Selective Charge Transfer

Shreetu Shrestha[1], Mingxing Li[1], Suji Park[1], Xiao Tong[1], Donald DiMarzio[2], Mircea Cotlet[1]

[1]Center for Functional Nanomaterials, Brookhaven National Laboratory, Upton, NY 11973, USA

[2]Northrop Grumman Corporation, One Space Park, Redondo Beach, California 90278, USA

Correspondence to cotlet@bnl.gov, sshrestha@bnl.gov

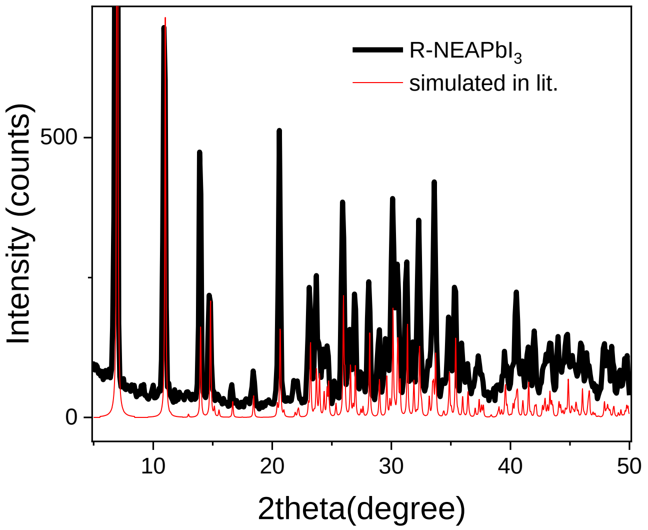


**Fig. 1** Powder X-ray diffraction of R-NEAPbI$_3$ synthesized (black) and simulated (red) in previous literature[1].

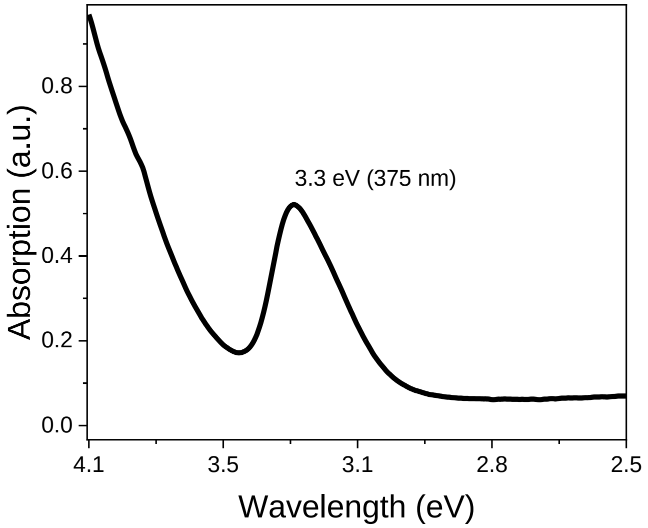


**Fig. 2** Linear absorption spectra of chiral perovskite R-NEAPbI$_3$

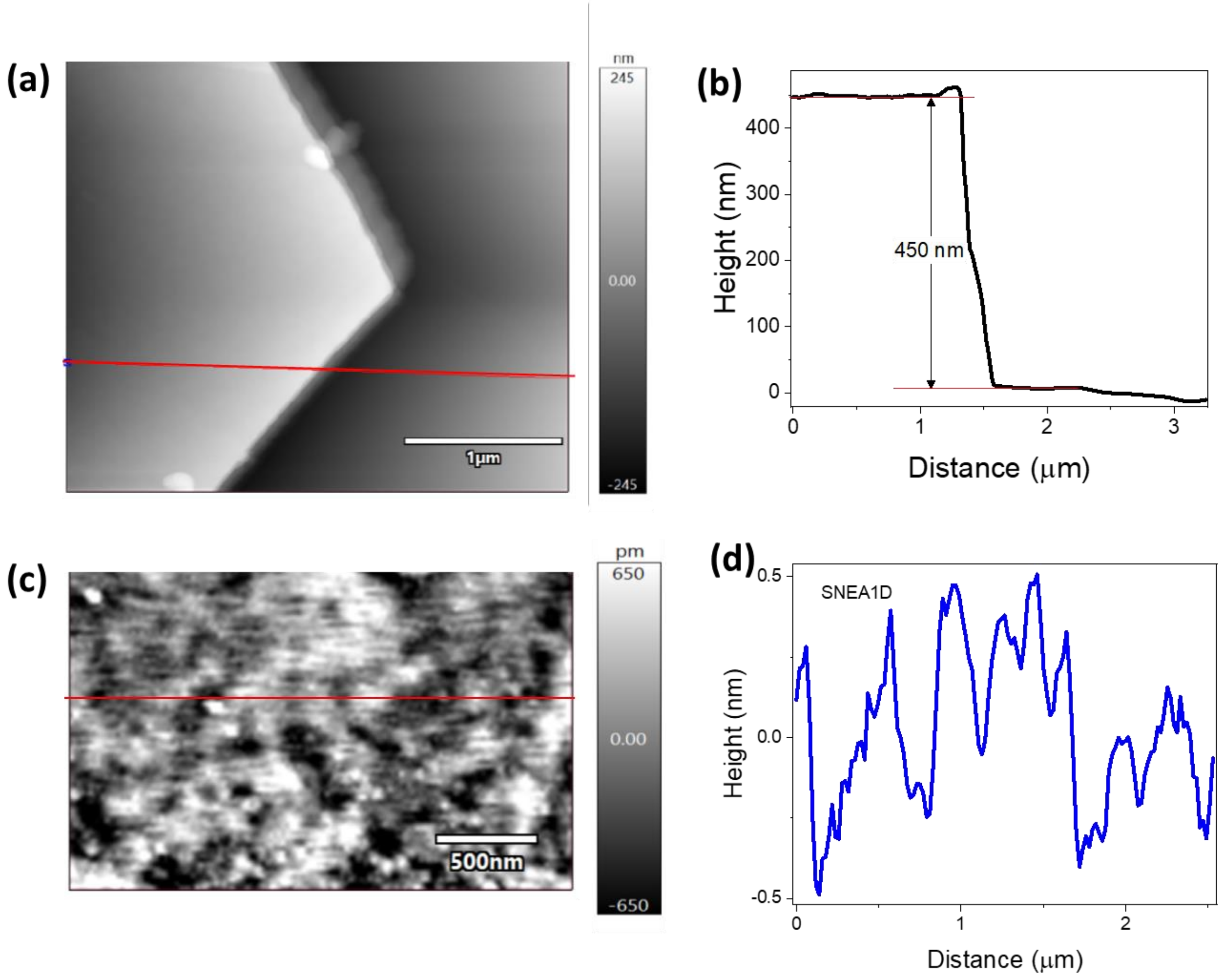


**Fig. 3** AFM of a chiral perovskite flake (a) image of a flake edge (b) flake height (c) zoomed in region of the flake (d) the surface roughness was ±0.5 nm.

(a)

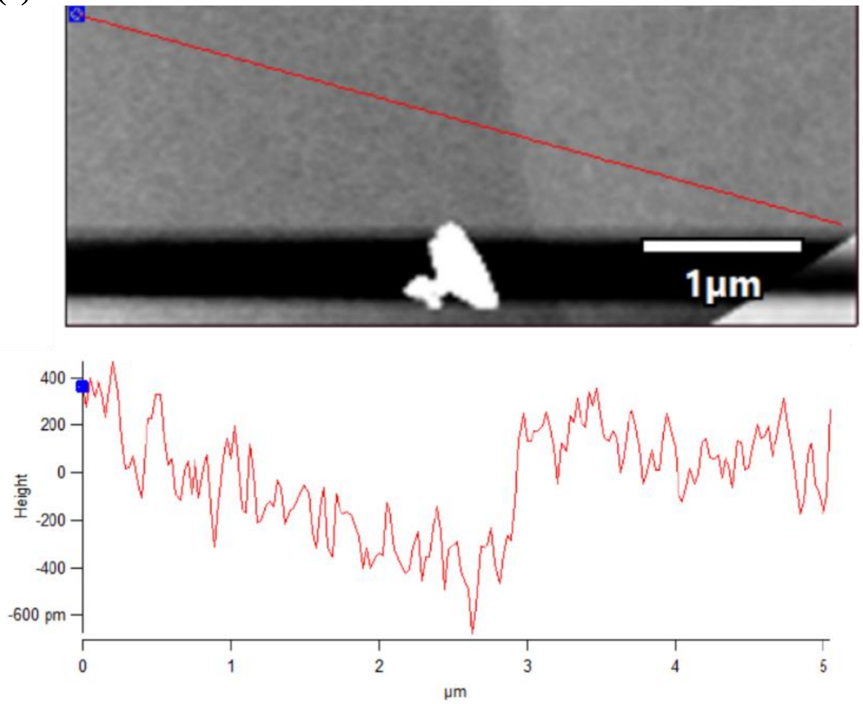


(b)

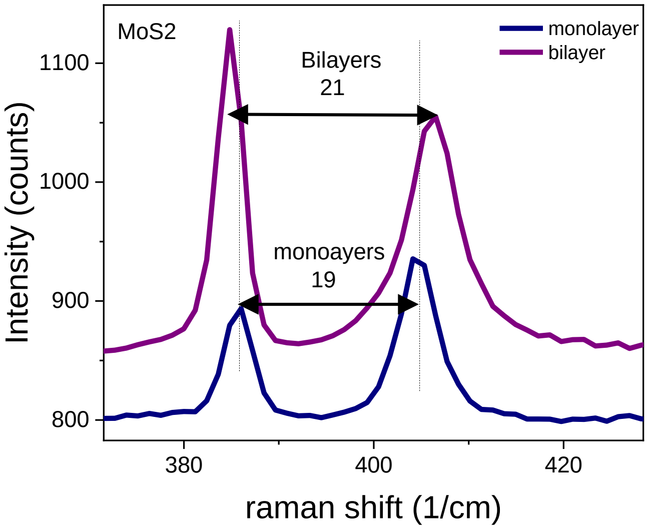


**Fig. 4** Identification of monolayer $MoS_2$ using (a) AFM which shows a height of < 1000 pm and (b) Raman spectroscopy where monolayers show frequency difference between $E^1_{2g}$ and $A_{1g}$ mode at 386 $cm^{-1}$ and 405 $cm^{-1}$ of less than 19 $cm^{-1}$

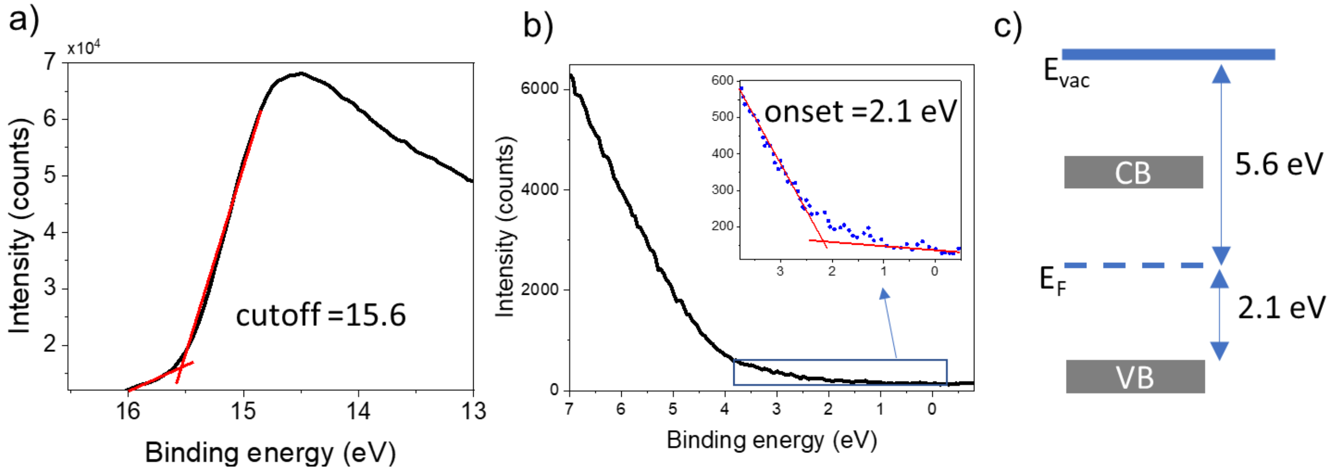


**Fig. 5** Ultraviolet photoelectron spectroscopy (UPS) of 1D chiral perovskite R-NEAPb$I_3$. The determination of (a) cutoff and (b) onset. (c) schematic of energy band

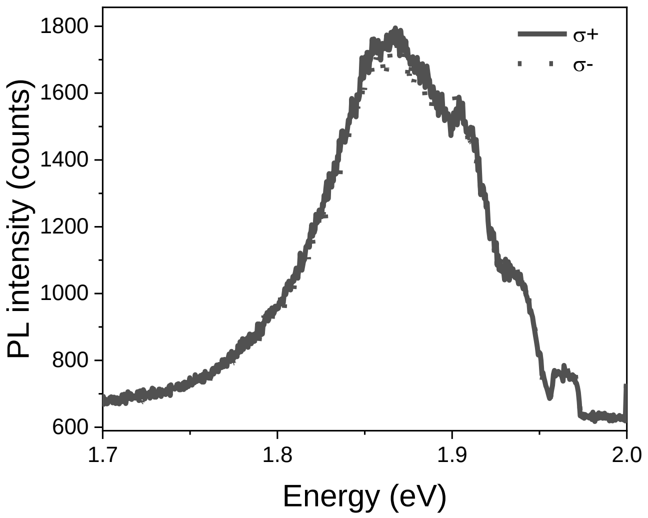


**Fig. 6** PL from heterostructures of S-NEAPb$I_3$/bilayer $MoS_2$ show negligible difference.

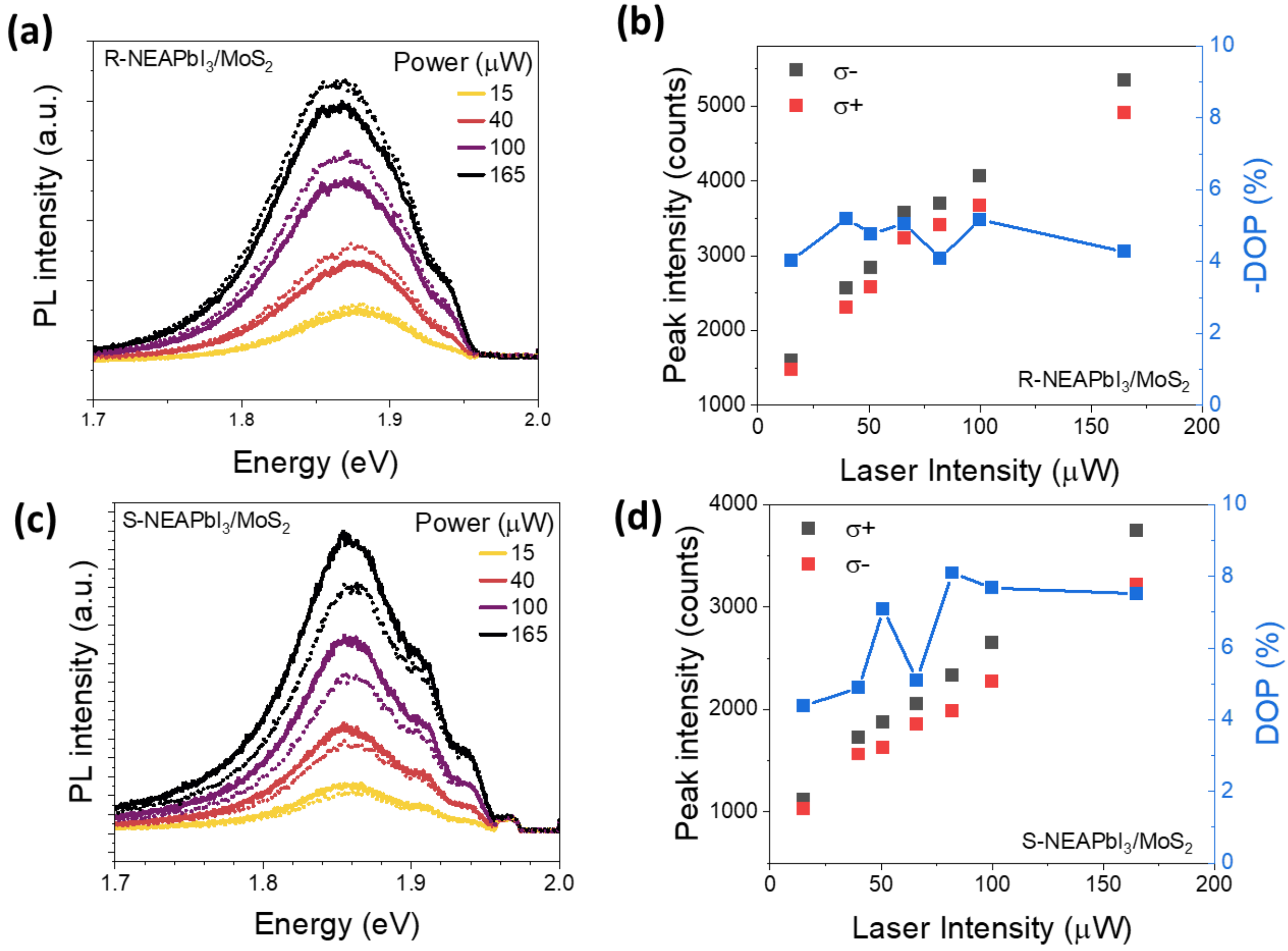


**Fig. 7** Laser intensity dependance. PL from R-NEAPb$I_3$/Mo$S_2$ (a) and S-NEAPb$I_3$/Mo$S_2$ (c) heterostructures at different laser intensities. The dotted line show σ- component while the solid lines show σ+. Peak PL intensity as a function of laser intensity of (b) R-NEAPb$I_3$/Mo$S_2$ and (d) S-NEAPb$I_3$/Mo$S_2$ heterostructures. The degree of polarization is shown on the right y-axis.

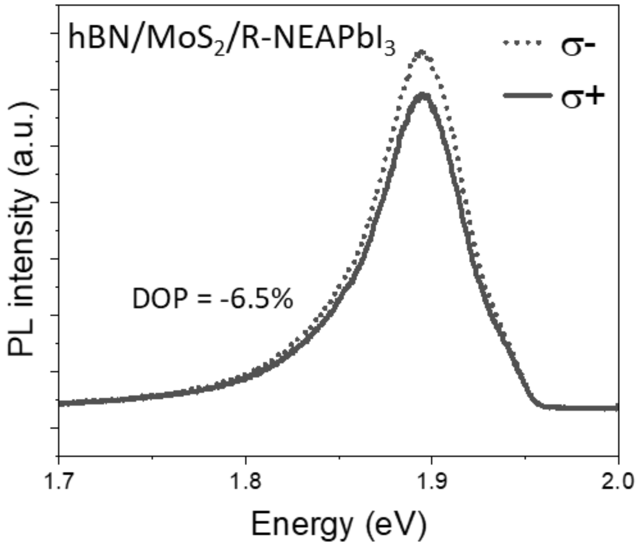


**Fig. 8**. Helicity dependent PL spectra of hBN/$MoS_2$/R-$NEAPbI_3$

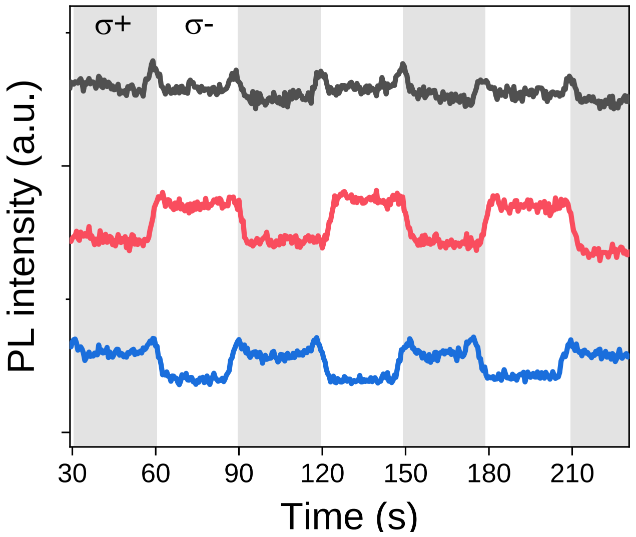


**Fig. 9** Time trace of PL intensity of $MoS_2$ monolayer (black), R-$NEAPbI_3$/$MoS_2$ (red) and S-$NEAPbI_3$/$MoS_2$ (blue) measured with an avalanche photodiode. The σ+ (grey region) and σ- components (white region) of the PL are selected by using a motorized rotation stage to rotate the quarter waveplate every 30 s. Small spikes in the PL intensity appear when the quarter waveplate is rotated because it passes through 0° where it is parallel with the liner polarizer as we rotate it from 45° to -45°. All measurements are performed with 100 µW 1.96 eV (633 nm) cw laser at room temperature.

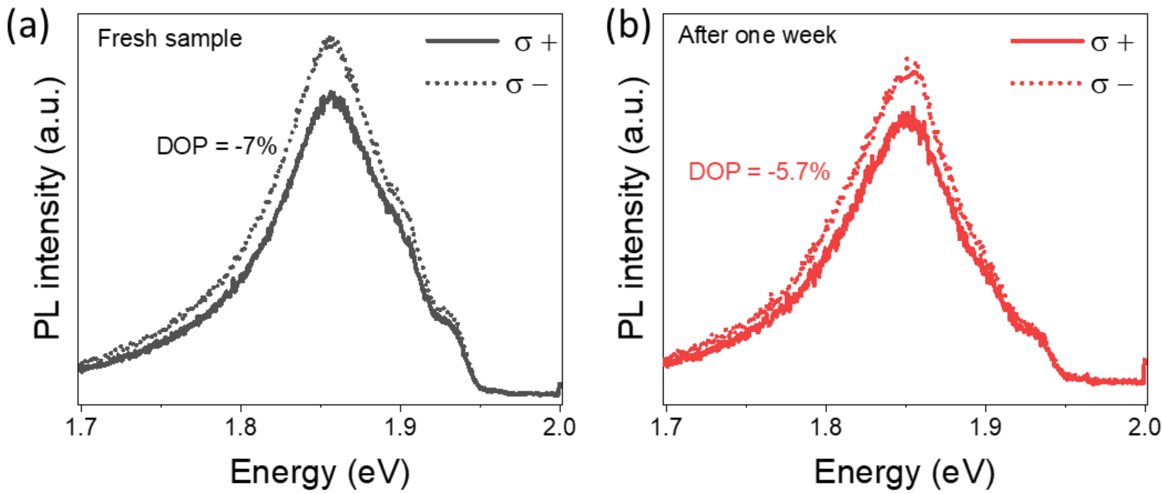


**Fig. 10** Helicity resolved PL of $MoS_2$/R-NEAP$bI_3$ heterostructures (a) fresh (b) after one week

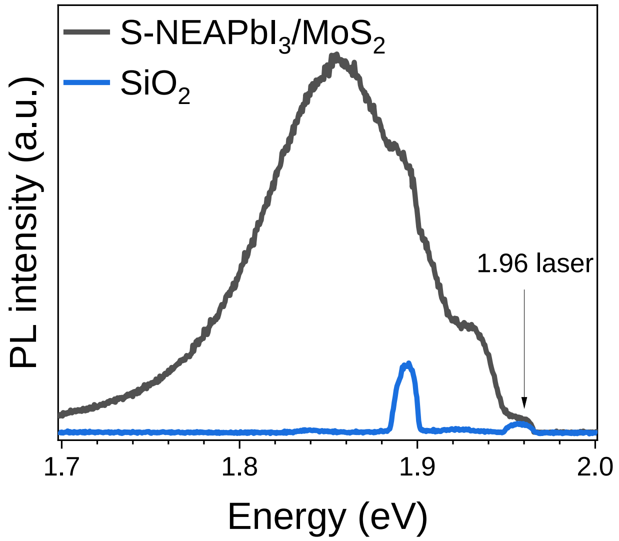


**Fig. 11** Polarization resolved PL from S-NEAP$bI_3$/$MoS_2$ heterostructure and $SiO_2$ substrate. The emission appears to have an additional peak at 1.89 eV. This signal was also observed from $SiO_2$ substrates measured under the same conditions. Therefore, we attribute the peak to scattered laser leaking in through the dichroic filter and long pass filter. The shoulder like feature at 1.93 eV is probably from the filter edge. This is further supported by data from heterostructures on hBN (Fig. 8). In this case, since the emission from the sample was high, the peak at 1.89 eV is absent while a slight shoulder can still be seen at 1.93 eV. More importantly, we also obtained a similar DOP of -6.5 % from hBN/$MoS_2$/R-NEAP$bI_3$ heterostructure (Fig. 8). This indicates that the additional peaks due to measurement artifacts does not influence our results and interpretation.

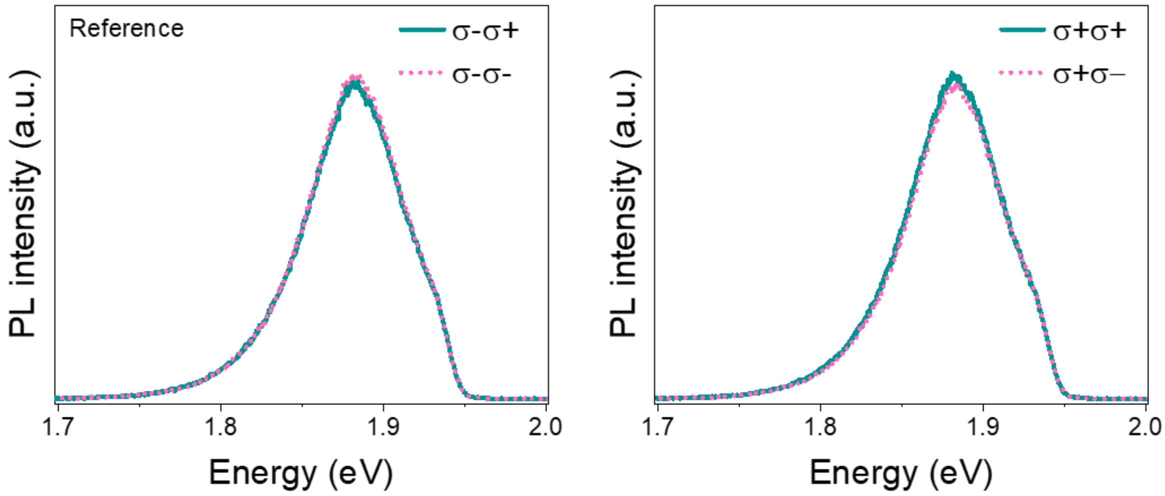


**Fig. 12** Polarization resolved PL from a non-chiral reference (Carboxyl Quantum Dots). To check our measurement system, we also measured a non-chiral sample (Carboxyl Quantum Dots from Ocean NanoTech) with emission at 1.86 eV as reference for which we obtained a negligible DOP of less than ± 1%.

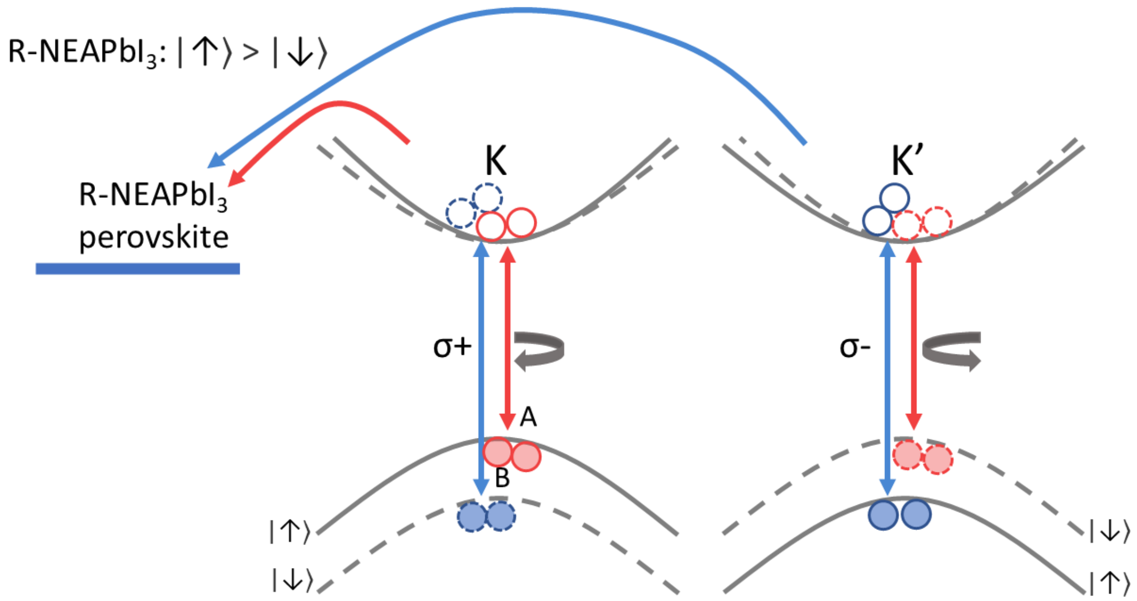


**Fig. 13** Schematics of charge transfer in RNEAPb$I_3$/Mo$S_2$ heterostructure. R-NEAPb$I_3$ preferentially selects spin up electrons (solid outlines) which in the K valley form A exciton and K’ valley form B excitons.

(a)

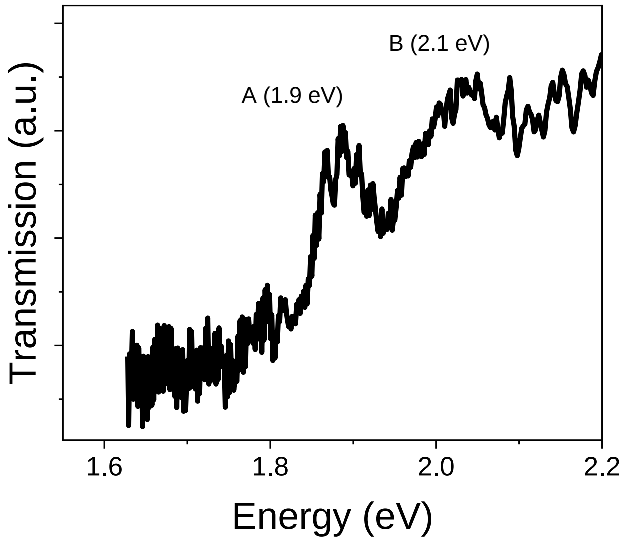


(b)

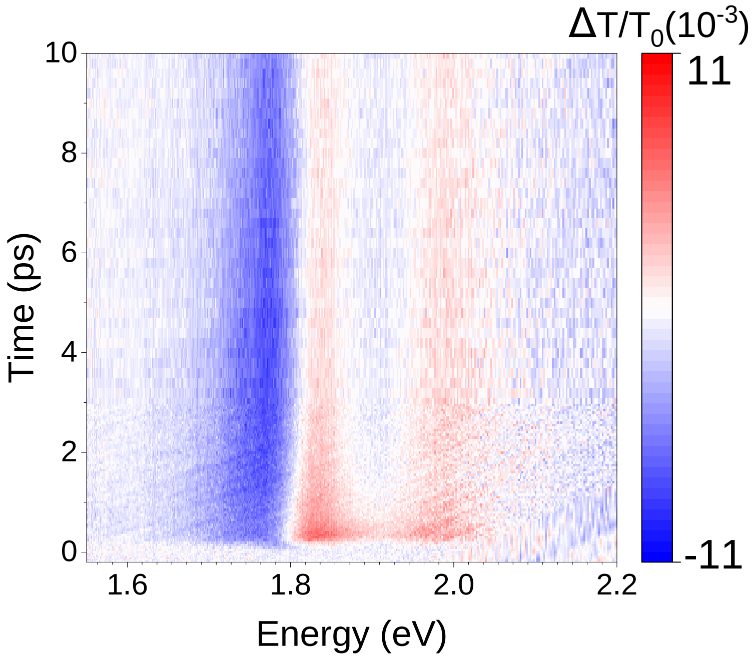


**Fig. 14** Monolayer $MoS_2$ transmission (a) steady state transmission (b) differential transmission

References

1 Jana, M. K. *et al.* 2015615 (The Cambridge Crystallographic Data Center, 2020).
